# PREVALENCE OF ZOONOTIC *CRYPTOSPORIDIUM SPP*. ISOLATES IN NJORO SUB-COUNTY, NAKURU COUNTY, KENYA

**DOI:** 10.21010/ajid.v15i2.2

**Published:** 2021-03-18

**Authors:** Essendi Miding’a Walter, Muleke Charles, Otachi Elick, Miheso Manfred, Kyule Domitila

**Affiliations:** 1 Egerton University, Department of Biological Sciences, P.O Box 536-20115, Egerton, Kenya; 2 Kenya Agricultural and Livestock Research Organisation, Food Crops Research Centre Njoro, Kenya; 3 Kenya Marine and Fisheries Research Institute(KMFRI), P.O Box 451-10230,Sagana, Kenya

**Keywords:** Cryptosporidiosis, Epidemiology, Prevalence, Zoonosis

## Abstract

**Background:**

There is no information on human and animal *Cryptosporidium* *spp*. in Njoro sub- county. The risk posed to humans and animals within the sub-county is therefore unknown.

**Materials and Methods:**

A total of 1476 animal and 378 human fecal samples were evaluated. Multivariate logistic regression was used to evaluate association between infection status and the predisposing factors. Results were expressed as odds ratio (OR) with a 95% confidence interval. Chi-square and Maentel–Haenszel tests were used to quantify relationships among variables.

**Results:**

Prevalence of *Cryptosporidium spp*. was 9.8% in humans, 10.8% in cows, 19.6% in sheep and 4.5% in goats. Prevalence in humans was significantly higher in females 12/37. Infection was highest in the elderly (27.27%), and significantly lower in adolescents and adults at 8.66% and 9.59%, respectively. Goats had lowest overall parasitization at all levels, while sheep had the highest parasitization at levels (+1 and +2). Relatively, humans had the highest parasite counts +3 cases (1.5%).

**Conclusion:**

*Cryptosporidium spp*. is prevalent in Njoro sub-county and domestic animals are important reservoirs and a potential source of zoonosis in humans. Children, elderly and females are at increased risk of infection, especially during rainy season. The study recommends maintenance of proper sanitation when handling domestic animals, treatment of drinking water and use of alternative safer sources of water in order to reduce infection.

## Introduction

Cryptosporidiosis is a zoonotic gastrointestinal illness caused by *Cryptosporidium* spp. an infectious pathogen that lives in the intestines of humans and other mammals (Ibrahim, 2002). Cryptosporidiosis causes severe diarrhea in very young, old and immunocompromised humans and cattle (Chen *et al.*, 2002). It is responsible for 8-19% of diarrhea in developing countries (Fayer *et al.*, 2000; Tzipori and Ward, 2002), and by 2010, it had caused about 100,000 deaths globally (Lozano *et al.*, 2012). *Cryptosporidium* parasite continues to be a major threat to human health for two major reasons. First, the current methods of water purification are ineffective for its removal from the public water supply, and secondly, there is no effective therapy for Cryptosporidiosis (Abubakar *et al.*, 2007).

River Njoro is the main source of water to the Njoro Sub-county. This river is a crucial source of surface and ground water for communities and ecosystems in and surrounding the watershed, including Njoro Sub-county, Nakuru Municipality and Lake Nakuru (Jenkins, 2011). *Cryptosporidium* contamination in River Njoro was evident in the Sustainable Management of Rural Watersheds (SUMAWA) project study of 2005 (Jenkins, 2011), which reported patterns and sources of feacal pollution in River Njoro watershed, but did not make an effort to characterize the specific parasites in the water. However, there is no information on the level of contamination of this river by *Cryptosporidium* parasites. Therefore, the risk exposed to by the domestic animals and humans that depend on this river is unknown.

This study aimed at determining the epidemiology of zoonotic *cryptosporidium* spp. in Njoro Sub-county.

*Cryptosporidium spp*. is an oocyst-forming apicomplexan protozoan. There are three features of Cryptosporidium spp. which ensure a high level of environmental contamination and an increase in the likelihood of waterborne transmission. Firstly, they are responsible for disease in a broad range of hosts including man (DuPont *et al.*, 1995; Grinberg *et al.*, 2008). They have a low-infectious dose (10–30 oocysts) enhancing the possibility of infection in healthy immunocompetent people (DuPont *et al.*, 1995; Okhuysen *et al.*, 1999). They may shed 108-109 oocysts in a single bowel movement and excrete oocysts up to 50 days after cessation of diarrhea (Jokipii *et al.*, 1986; Chappell *et al.*, 1996). Secondly, their oocysts are small in size and environmentally robust (Hsu *et al.*, 2001; Robertson and Gjerde, 2001). Thirdly, they are insensitive to the normal disinfectants commonly used in the water industry (Reinoso *et al.*, 2008; Shields *et al.*, 2008). The warm and humid environment, contaminated water, poor sewage disposal, overcrowding, poverty, illiteracy and limited health services all contribute to the transmission and persistence of the parasite in developing countries (Snelling *et al.*, 2007).

Cryptosporidiosis infections are spread from animal-to-animal by the fecal-oral route, usually when animals are housed together in an overcrowded environment (Xiao *et al.*, 2004). Contamination of udders and water supplies by feaces is another common source of transmission in livestock. Other zoonotic transmissions have been confirmed from pets, farm animals and by accidental infection of veterinary workers. There is data on human-to-human transmission between family members, sexual partners, children in daycare centers, and hospital patients and staff (Current and Garcia, 1991). Food handled by an infected person or food grown in soil fertilized with manure are sources of food-borne Cryptosporidiosis. However, contaminated water represents the major source of infections for humans (Hooda *et al.*, 2000). Pathogen genotyping efforts based on the analysis of 18S rDNA has identified 9 Cryptosporidium genotypes: human, bovine, pig, cat, mouse, dog, monkey, ferret and masurpial (Ramirez *et al.*, 2004).

## Materials and Methods

### Study area

This study was conducted in River Njoro watershed, in Nakuru county which lies between 0°30’S and 35° 20’ E (Owino, 2013). River Njoro originates in the Eastern Mau and covers about 50 km in length, and an estimated source area of about 270 km^2^. River Njoro watershed comprises of forested, agricultural lands and urban settlements, and terminates in Lake Nakuru (Owino, 2013)

**Figure 1:**
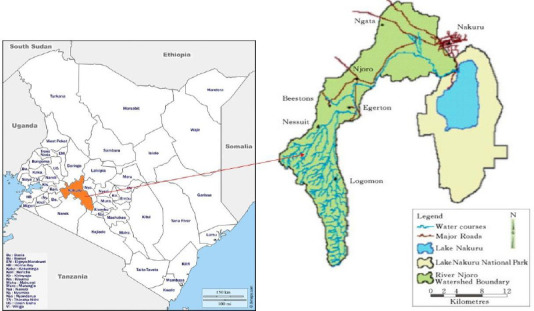
Map of location of Njoro river watershed. Adopted from (Owino, 2013).

### Study Design

Complete Randomized Design was used in this study to give each region sampled within the Njoro Sub-county an equal chance of being included in this study. Purposive sampling design was employed when sampling humans where only patients who showed signs of diarrhea were sampled because they were more likely to be infected with cryptosporidiosis. Hospitals and homesteads were randomly selected. Inclusion criteria involved only those individuals who resided within the Njoro river watershed.

### Samples

The units of sampling were humans and domestic animals, while the samples used in this study were human and animal fecal matter. A total of 1854 randomly collected fecal samples; 378 from human, 1000 cows, 388 sheep and 88 from goats were evaluated.

### Sample collection

For humans, clinical samples for this study were collected as 10 g of feces/stool samples from 378 diarrheic patients from public health facilities within Njoro Sub-county, where prevailing conditions favor presence of clinical Cryptosporidiosis. Accompanying information such as age, sex and source of water used by the patient were directly collected from patients through direct interview.

Animal samples were collected as 10 g of dung taken from each of the 1000 cows, 88 goats and 388 sheep obtained from each of the clustered households within Njoro Sub County. The animal owners were interviewed on the source of water given to the animals. The collected samples were transported to the Biological Science Laboratory at Egerton University, and refrigerated at 4°C.

### Identification of *Cryptosporidium* spp. in fecal samples

All the fecal samples were tested for the presence of oocysts of *Cryptosporidium* spp. using the Ziehl –Neelsen staining technique as described by Henriksen and Pohlenz (1981). The fecal smears were prepared on a microscope slide, air dried and fixed with methanol for 5 min. Fixed smears were stained with dilute carbol fuchsin (1:10) for 3–5 min, and washed with tap water. Smears were decolorized using acid alcohol, then counterstained with 0.5 % malachite green solution for 1 min. Smear slides were air dried and then examined under the microscope at x400 magnification. *Cryptosporidium* spp. oocysts appear as pink to red, spherical to ovoid bodies against a green to purple background. Samples were considered positive if at least one morphologically distinct *Cryptosporidium* spp. oocyst was observed.

### Data analysis

Data Analysis was done using R-Studio version 1.2.5019 (R Core Team (2019). Prevalence of *cryptosporidium* was analyzed using summary statistics and presented in tables and graphs. Multivariate logistic regression using the status of the humans and animals (infected or non-infected) as the dependent variable, and the independent variables were the sources of water and predisposing in humans (Age, sex and season). The results were each expressed as an odds ratio (OR) with a 95% confidence interval (CI 95%). To study the possible association between those variables in which there is no temporal relationship and which do not allow causal relationships to be established. Chi square (χ2)-test of association, was used to measure the relationship among variables in which there was no temporal relationship could be identified. Maentel–Haenszel test was used for analysis of those variables with more than two categories in order to examine their linear trend. Two-sided P < 0.05 was used as the level of significance.

### Ethical considerations and approval

This study was carried out after approval by research and ethical committee of Egerton University (EUREC/APP/093/2019; Date: 4/3/2020). Permission was also obtained from Hospitals and abattoirs. Informed consent of the participants was also obtained.

## Results

### Prevalence of Cryptosporidium spp. in humans and domestic animals

Results show that 37 of the 378 (9.8%) human samples were positive, 108 out of 1000 (10.8%) cows’ samples were positive, a total of 76 out of 388 (19.6%) sheep samples were positive and (76/388) in sheep and only 4 out of 88 (4.5%) goat samples were positive for *Cryptosporidium* oocysts (Table 1).

**Table 1:** Prevalence of *Cryptosporidium* in humans with sex, age and water source

		Total (N)	Positive (n)	(n/N (%)	Odd ratio	95% Conf. Interval	chi2	Pr > chi2
Sex	Male	153	12	7.84%	0.25	0.08 - 0.76	11.717	0.0006***
	Female	188	25	13.30%	0.333	0.11 - 0 .98		
Age	0-10yrs	203	23	11.33%	0.077	0.02 - 0.37	16.674	0.0018**
	11yrs-20yrs	54	4	8.66%	0.333	0 .11 - 0.98		
	21yrs-40yrs	73	7	9.59%	0.6	0.13 - 2.69		
	41yrs-70yrs	11	3	27.27%	0.2	0.61 - 0.65		
Water Source	River	237	30	12.65%	0.333	0.11 - 0.98	16.865	0.0006***
	Tap	76	5	6.58%	1	0		
	Borehole	20	2	10%	0.25	0.08 - 0.76		

Significance codes: 0

’***’ 0.001

‘**’ 0.01 ‘*’ 0.05 ‘.’ 0.1 ’ ’ 1

### Prevalence of *Cryptosporidium* infection by age and sex

To analyze prevalence of *Cryptosporidium* by sex, 341 cases; 153 males while 188 females were evaluated and 37 cases were found to be positive of which 12 were male while 25 were female. *Cryptosporidium* prevalence was significantly higher in females than in male (*χ2, P* = 0.0006). The risk of infection was found to be significantly higher in females than in males (OR: 0.25, CI 95%: 0.08 - 0.76). When evaluating the relationship between prevalence and age, 378 cases were studied. Results obtained show that prevalence of *Cryptosporidium* was significantly different between all age groups. Prevalence was highest in older individuals aged between 41 – 70 years at 27.27% and significantly lower in adolescents (11 – 20) years and adults aged 21-40 years who recorded 8.66% and 9.59%( *χ2, P* = 0.0018), respectively (Table 1).

### Prevalence of *Cryptosporidium* infection by water source

**Figure 2:**
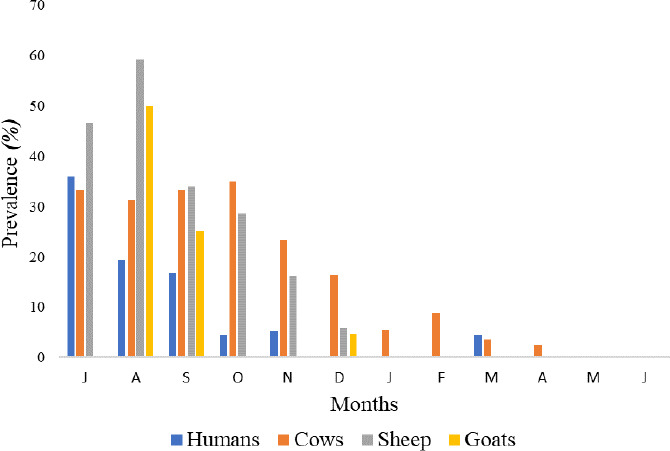
Relationship between Prevalence of *Cryptosporidium* spp. in humans and Domestic animals and season.

A total of 341 human samples from donors who sourced water from three water sources, Njoro river 237, boreholes 28, and Tap 76 were evaluated. The effect of water sources on prevalence was evaluated and results showed that individuals who sourced water from Njoro river recorded significantly higher prevalence at 12.65% (*χ2, P =* 0.0006), while tap water source was associated with the least prevalence of 6.58% (*χ2, P =* 0.0006) (Table 1). When the effect of season on relationship between the water source and prevalence was assessed, it was apparent that the prevalence was highest during the rainy season with only 1 case 9.1% in river Njoro and no case for boreholes and tap water sources occurring during the dry season (Figure 2).

### Prevalence of *Cryptosporidium* in humans and domestic animals by season

When association of *Cryptosporidium* in humans and domestic animals was compared, it was highest in sheep in wet/rainy months (July – November) and lowest in cows in dry season (December – June). Cows had relatively high prevalence of *Cryptosporidium* throughout the year except in May and June where no cases were reported. Goats had the least prevalence of all the domestic animals with cases reported in only three months (August, September and December). For humans, *Cryptosporidium* monthly prevalence ranged between 0% (April, May, June) and 35.9% in July. There was a significant difference between prevalence of *Cryptosporidium* in wet and dry seasons (p = 0.0842). The prevalence of *Cryptosporidium* was relatively higher in wet and rainy months, peaking in July, than in dry seasons where prevalence was lowest in December, January and February (Figure 2).

### *Cryptosporidium* Parasite counts in humans and Domestic animals

In order to evaluate parasite concentration levels in humans and domestic animals, fecal samples from 378 humans, 1000 cows, 388 sheep and 88 goats were analyzed. Parasite count was significantly different in humans and domestic animals. Goats recorded lowest overall parasite counts than all the domestic animals for all levels (+1, +2 and +3), while sheep had the highest parasitization level +1 and +2. Relatively, humans had the highest parasite counts +3 cases 6 (1.5%) than all the domestic animals tested.

Parasite count level +1 was the most common type for all animals with highest number of cases in each case, followed by parasite count level +2 which was observed in tested humans and animals but at lower incidence, while parasite count level +3 was the least with cases observed in humans and sheep only.

**Figure 3:**
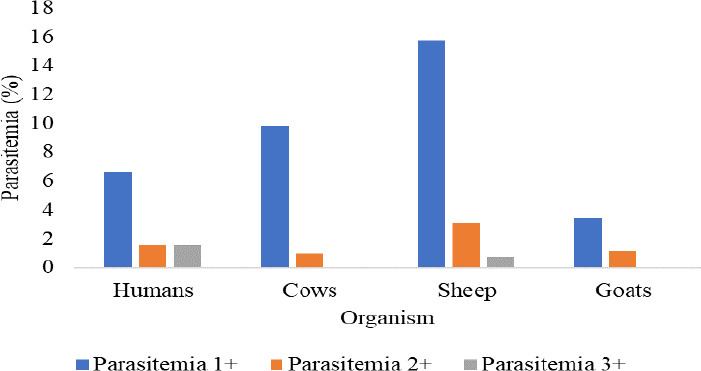
*Cryptosporidium* parasite counts in humans and domestic animals

## Discussion

This study elucidated the prevalence of Cryptosporidosis in human and domestic animals that source water from Njoro river watershed and surrounding water sources; boreholes and taps. Prevalence was higher in the older individuals aged between 41-70 years and children aged between 0 – 10 years, and lowest in adolescents aged between 11- 20 and adults 20-40 years. These results are similar to those reported in other studies (Hlavsa *et al.*, 2012; Nahed and Ghoneim, 2017 and Tombang *et al.*, 2019), where incidence and prevalence was found to be highest in children and old age individuals. In this study, the infection rate observed (9.8%) was higher than the prevalence previously reported by Mbae (2006), 4% in children in urban informal settings. However, in this study, we evaluated the effect of zoonotic *Cryptosporidium* spp. in a rural setting dependent on a river watershed, and involved all age groups while Mbae (2006) study was done in an informal urban setting and was restricted to children. Higher prevalence rates have also been reported in other rural settings in developing countries like Gambia (12%) (Hossain, 2019), Rwanda (10.4%) (Angus, 2013) and Nigeria (5.3%) (Reinthaler, 2007). The high prevalence of *Cryptosporidium* spp. in rural areas in developing countries may be attributed to lack of access to clean drinking water, cohabitation with animals, and inadequate hygiene as is characteristic of many resource poor settings (Mbae, 2006). However, the reported infection rate is higher than the reported *Cryptosporidium* prevalence in developed countries such as USA where prevalence ranges from 0.0% to 2.2% (Hlavsa *et al.*, 2012). High infection rate in the elderly at 41-70 years, maybe as a result of compromised immunity in old age, or continuous exposure to contaminated food and water. The high prevalence in infants at 0 – 11 years may be due to exposure to contaminated water and food during the weaning period, at home and school (Hlavsa *et al.*, 2012). Lower rates of infection in adolescents and adults could be due to boosted immunity due to secondary exposure. Chappell (2009), linked higher levels of anti-*Cryptosporidium* IgG antibody to reduced chances of *Cryptosporidium* oocyst infection and illness in adults.

Significantly higher prevalence was experienced in females (13.30%) than in males (7.84%) (P=0.000195***), probably because of increased exposure of females to untreated water compared to their male counterparts. This is in line with the results obtained in Uganda by Tombang *et al.*, (2019) who recorded higher prevalence in females (3.3%) than in males.

In this study, 97.3% of *Cryptosporidium*-positive in humans and 79.6% cows and all cases in sheep and goats were observed during the rainy season; between July and December. This finding is in agreement with numerous past studies which also found positive association between high *Cryptosporidium* infection and rainy season globally (Areeshi, 2002; Das, 2006; Perreira, 2008; and Adegbola, 2014). This high prevalence may be as a result of use of surface run off water which is often contaminated with human and or animal feces for domestic purposes and contamination of boreholes and other water sources with surface water. On the Contrary, Mbae *et al.*, 2006 reported that *Cryptosporidium* infection in children in urban settings of Nairobi, Kenya, was highest in dry months, compared to rainy months (Mbae, 2006). This has been associated with water shortage in urban areas during dry seasons leading to drinking and domestic use of contaminated water.

## Conclusion

This study established Cryptosporidiosis as an important disease in Njoro river watershed in Nakuru county of Kenya. The study highlighted domestic animals as an important reservoir for *Cryptosporidium* *spp*. and a potential source of zoonosis of this infection in humans. Children, the elderly and females are at an increased risk of infection from *Cryptosporidium* *spp*; especially during the rainy season. The study recommended maintenance of proper sanitation when handling domestic animal fecal matter. Treatment of drinking water using chemicals such as chlorine or boiling of water could be useful in reducing transmission. Use of alternative safer sources of water such as tap water and well-sealed boreholes may reduce over reliance on surface water which is the main source of infection.

**List of Abbreviation:**
SUMAWA: : Sustainable Management of Rural Watersheds
OR: : Odds ratio
CI: : Confidence Interval
IgG: : Immunoglobulin G

## References

[1] Abubakar I, Aliyu S. H, Arumugam C, Usman N. K, Hunter P.R (2007). Treatment of Cryptosporidiosis in immunocompromised individuals:Systematic review and meta-analysis. British Journal of Clinical Pharmacology.

[2] Adegbola RA, D. E (1994). *Cryptosporidium* infection in Gambian children less than 5 years of age. Journal of Tropical Medicine and Hygiene.

[3] Angus. (2013). Cryptosporidiosis in man domestic animals and birds Journal of Clinical Microbiology.

[4] Areeshi M. D. W (2008). *Cryptosporidium* species causing acute diarrhoea in children in Antananarivo Madagascar. Annals of Tropical Medicine and Parasitology.

[5] Chappell O. P (2009). Infectivity of *Cryptosporidium parvum* in healthy adults with pre-existing anti-C parvum serum immunoglobulin G. Tropical Medicine;Hygiene.

[6] Chen X. M, Keithly J. S, Paya C. V, La Russo N. F (2002). Cryptosporidiosis. The New England Journal of Medicine.

[7] Connelly JT N.S.-W. (2008). Human pathogenic *Cryptosporidium* species bioanalytical detection method with single oocyst detection capability. Anal Bioanal Chem.

[8] Current W.L, Garcia L.S (1991). Cryptosporidiosis. Clinical Microbiology.

[9] Das R. S (2006). Molecular characterization of Cryptosporidium spp. from children in Kolkata India. Journal of Clinical Microbiology.

[10] DuPont H. L, Chappell C. L, Sterling C. R, Okhuysen P. C, Rose J. B, Jakubowski W (1995). The infectivity of *Cryptosporidium parvum* in healthy volunteers. The New England Journal of Medicine.

[11] Fayer R, Morgan U, Upton S. J (2000). Epidemiology of *Cryptosporidium*:transmission, detection and identification. International Journal of Parasitology.

[12] Grinberg A, Lopez-Villalobos N, Pomroy W, Widmer G, Smith H, Tait A (2008). Host-shaped segregation of the *Cryptosporidium parvum* multilocus genotype repertoire. Epidemiology and Infection.

[13] Google. (n.d.) Njoro sub county.

[14] Henriksen S. A, Pohlenz J.F.L (1981). Staining of *Cryptosporodia* by modified Ziehl- Neelsen technique:a brief communication. Acta Veterinaria Scandinavica.

[15] Hlavsa C. M, Painter E. J, &Collier A. S (2012). Cryptosporidiosis Surveillance —United States, 2011–2012. Morbidity and Mortality Weekly Report (MMWR).

[16] Hossain MJ, S. D (2019). *Cryptosporidium* infection in rural Gambian children:Epidemiology and risk factors. PLoS Neglcted Tropical Diseases.

[17] Hooda P. S, Edwards A. C, Anderson H.A, Miller A (2000). A review of water quality concerns in livestock farming areas. Science of the Total Environment.

[18] Hsu B. M, Huang C, Pan J. R (2001). Filtration behaviors of *Giardia* and *Cryptosporidium*-ionic strength and pH effects. Water Research.

[19] Ibrahim A. H (2002). Prevalence of intestinal parasites among school children in Deir-El-Balah town in Gaza strip Palestine. Annals of Saudi Medicine.

[20] Jenkins M. W (2011). Gross feacal pollution of a rural watershed in Kenya:research identifying cattle as a major source in the River Njoro Watershed. Research Brief 08- 01-SUMAWA.

[21] Lozano R, Naghavi M,  Foreman K, Lim S, Abraham J, Adair T, Mohammad A, Miriam A (2012). Global and regional mortality from 235 causes of death for 20 age groups in 1990 and 2010:a systematic analysis for the Global Burden of Disease Study 2010. Lancet.

[22] Mbae E. M (2006). Genetic Diversity of *Cryptosporidium* in Children in an Urban Informal Settlement of Nairobi Kenya. The American Society of Tropical Medicine and Hygiene.

[23] Nahed H, Ghoneim M. A (2017). Prevalence and Molecular Epidemiology of *Cryptosporidium* Infection in Calves and Hospitalized Children in Egypt. Research Journal of Parasitology.

[24] Owino Z. G (2013). Effects of land use and management on aggregate stability and hydraulic conductivity of soils within River Njoro Watershed in Kenya. International Soil and Water Research.

[25] Pereira A. E.-Z (2002). Intra-familial and extra-familial risk factors associated with *Cryptosporidium parvum* infection among children hospitalized for diarrhea in Goiania Goias, Brazil. Journal of Tropicalk Medicine.

[26] Ramirez N. E, Ward L. A, Sreevatsan S (2004). A review of the biology and epidemiology of Cryptosporidiosis in humans and animals. Microbes Infection.

[27] R Core Team (2019). (2019 October 24) R:A language and environment for statistical computing.

[28] Reinthaler F (2007). Cryptosporidiosis in Ogun State southwest Nigeria. Tropical Medicine.

[29] Reinoso R, Becares E, Smith H. V (2008). Effect of various environmental factors on the viability of *Cryptosporidium parvum* oocysts. Journal of Applied Microbiology.

[30] Robertson L. J, Gjerde B (2001). Occurrence of parasites on fruits and vegetables in Norway. Journal of Food Protection.

[31] Okhuysen P. C, Chappell C. L, Crabb J. H, Sterling C. R, DuPont H. L (1999). Virulence of three distinct *Cryptosporidium parvum* isolates for healthy adults. The Journal of Infectious Diseases.

[32] Shields J. M, Hill V. R, Arrowood M. J, Beach M. J (2008). Inactivation of *Cryptosporidium parvum* under chlorinated recreational water conditions. Journal of Water and Health.

[33] Snelling J. W, Lihua X, Guadalupe O, Colm J, Lowery I, John E, Juluri R, Stephen S. B, Cherie M, Paul J, Rooney M. M, Fiona K, Jiru X, James S. G (2007). Cryptosporidiosis in developing countries. Journal of Infections in Developing Countries.

[34] Tombang A, Ambe, Bobga T (2019). Prevalence and risk factors associated with cryptosporidiosis among children within the ages 0–5?years attending the Limbe regional hospital, southwest region, Cameroon. BMC Public Health.

[35] Tzipori S, Ward H (2002). Cryptosporidiosis:biology pathogenesis and disease. Microbes and Infection.

[36] Xiao L, Escalante L, Yang C, Sulaiman I, Escalante A. A, Montali R. J, Fayer R, Lal A. A (1999). Phylogenetic analysis of *Cryptosporidium* parasites based on the small-subunit rRNA gene locus. Applied and Environmental Microbiology.

